# The rate of microtubule breaking increases exponentially with curvature

**DOI:** 10.1038/s41598-022-24912-0

**Published:** 2022-12-03

**Authors:** Stanislav Tsitkov, Juan B. Rodriguez, Neda M. Bassir Kazeruni, May Sweet, Takahiro Nitta, Henry Hess

**Affiliations:** 1 Columbia University, 351L Engineering Terrace, MC 8904, 1210 Amsterdam Avenue, New York, NY 10027 USA; 2 Applied Physics Course, Faculty of Engineering, Gifu University, Gifu, 501-1193 Japan

**Keywords:** Biomedical engineering, Motility, Nanoscale biophysics

## Abstract

Microtubules, cylindrical assemblies of tubulin proteins with a 25 nm diameter and micrometer lengths, are a central part of the cytoskeleton and also serve as building blocks for nanobiodevices. Microtubule breaking can result from the activity of severing enzymes and mechanical stress. Breaking can lead to a loss of structural integrity, or an increase in the numbers of microtubules. We observed breaking of taxol-stabilized microtubules in a gliding motility assay where microtubules are propelled by surface-adhered kinesin-1 motor proteins. We find that over 95% of all breaking events are associated with the strong bending following pinning events (where the leading tip of the microtubule becomes stuck). Furthermore, the breaking rate increased exponentially with increasing curvature. These observations are explained by a model accounting for the complex mechanochemistry of a microtubule. The presence of severing enzymes is not required to observe breaking at rates comparable to those measured previously in cells.

## Introduction

The mechanics of cytoskeletal filaments, such as microtubules and actin filaments, is of continuing interest not only in cell biology and biophysics^1^, but also in nanotechnology^2^. Due to their structural complexity, the description of their elastic properties has proven to be a significant challenge^3–6^. A related but separate point of interest is the fracture behavior of cytoskeletal filaments: what levels of stretching or bending lead to breaking of the filament?

For actin filaments, the tensile strength was found to be 440 ± 120 pN (mean ± S.D.)^7^, while a knotted filament broke when the diameter was reduced to 0.36 ± 0.1 μm (mean ± S.D.)^8^. For microtubules, the tensile strength should be higher due to the larger number of protofilaments but has not been directly measured. Optical trap measurements observed rupture forces of only 3 ± 2 pN (mean ± S.D.), but attributed these low values to photodamage to the fluorescently labeled tubulins under excitation light^9–11^. The work of Waterman-Storer and Salmon provided first insights into microtubule breaking resulting from bending^12^: breaking of seven microtubules in newt lung cells occurred at an average curvature of 1.4 ± 0.3 μm^−1^. Odde et al.^13^ observed 24 breaking events of microtubules with a total length of 3 mm in fibroblasts, and determined an average breaking curvature of 1.5 ± 1.0 μm^−1^ (mean ± S.D.), significantly larger than the typical curvature of the microtubules which was approximately exponentially distributed with a mean of 0.39 μm^−1^ and a standard deviation of 0.53 μm^−1^ (n = 858). The average rate of breaking events per length of microtubule and time of 2.3 mm^−1^ min^−1^ was found to be physiologically significant. However, as Odde et al. point out, microtubule breaking in cells can result from curvature-dependent alterations in the population of stabilizing microtubule-associated proteins (MAPs), or curvature-dependent activation of microtubule-severing proteins (e.g. Katanin)^14^, thereby obscuring purely mechanical failure of the microtubule lattice. Recent work by Kabir et al.^15^ observed the breaking of microtubules in vitro as they were attached to a deformable substrate via surface-adhered kinesin motors. The frequency of microtubule breaking (per length of microtubule observed) increased from zero at a radius of curvature of 0.35 μm to 373 ± 3 mm^−1^ at a radius of curvature of 0.03 ± 0.004 μm as the substrate was slowly compressed over 2 min. What is missing from the discussion is a grounding of filament breaking in mechanochemistry^16,17^, that is a recognition that the occurrence of the intermolecular bond rupture events leading to the filament breaking depends on both, the applied stress and the duration of the stress application.

Here, breaking of paclitaxel-stabilized microtubules (paclitaxel was previously known as taxol) is studied in vitro by observing a large number of microtubules for an extended time as they glide over surface-adhered kinesins and analyzing the rare breaking events. We observed a total of 116 breaking events as we acquired over 190,000 images of microtubules in total. Only 5 of those breaking events occurred during smooth gliding, whereas 111 events occurred when the tip of the gliding microtubule was pinned and the fishtailing or spiraling of the microtubule induced strong bending^18–20^. The breaking events are then analyzed in the framework of mechanochemistry, which accounts for the relationships between time, force and molecular reactions^16,17^.

## Results

We observed the gliding of fluorescently labeled, paclitaxel-stabilized microtubules on full-length kinesin-1 physisorbed to casein-coated glass surfaces at a kinesin density of 500 μm^−2^ (determined by landing rate measurements^21,22^ as described in Bassir Kazeruni et al.^23^) and an ATP concentration of 1 mM resulting in a gliding velocity of 860 ± 50 nm/s. By imaging the microtubules every 2 s with an exposure time of 0.2 s, breaking events could be directly observed and manually counted.

**Figure 1:**
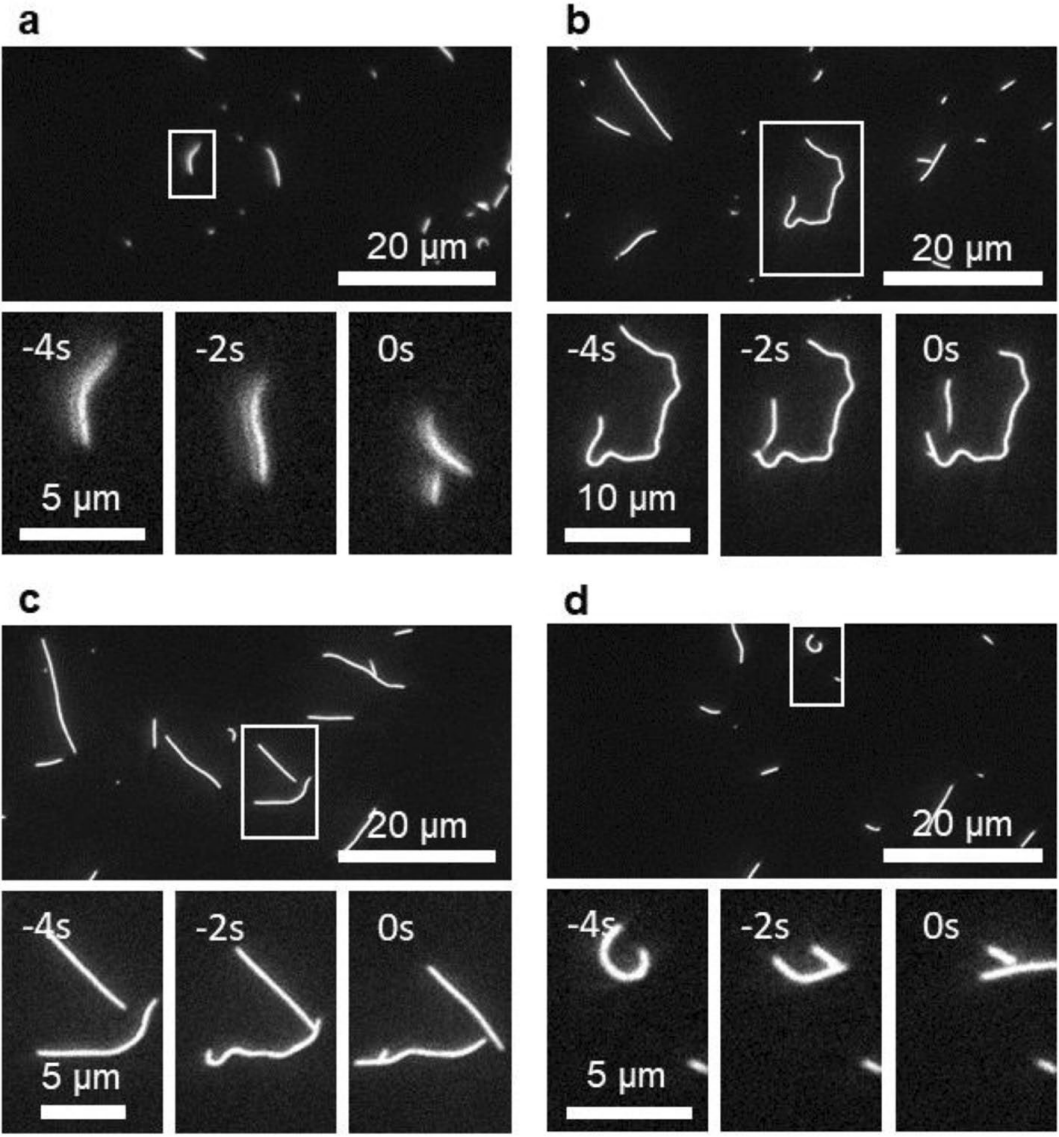
Fluorescence microscopy images of microtubule breaking. (**a**, **b**) Breaking while smoothly gliding. (**c**, **d**) Breaking while pinned.

Two types of breaking events can be distinguished: breaking while smoothly gliding (5 events, Fig. 1a, b) and breaking while pinned (111 events, Fig. 1c, d). Breaking while pinned occurs when a microtubule gets stuck on the surface due to the presence of an obstacle (most probably a defective kinesin motor or a dust particle^18–20^) on the surface, bends, and breaks primarily at points of high curvature.

Breaking while smoothly gliding could result from the gradual weakening of a microtubule due to the repeated forces exerted by kinesin motors on the microtubule^1,24–26^, the photodamage experienced while the microtubule is in the field-of-view^9–11^, and the strain on the microtubule lattice at locations of high curvature^13,15^.

If a microtubule is pinned and the pinned tip is free to rotate, the force exerted by the kinesin motors attached to the microtubule tail will cause the leading segment to buckle and the microtubule will “spiral” around the attachment point^18,19^. If the pinned tip is not free to rotate, the force exerted by the motors attached to the tail will cause buckling a little further down from the tip. The highly curved microtubule will eventually detach from the motors and straighten, and then the process begins again, giving it a “fishtailing” motion^18,27^. This behavior will give rise to microtubule segments with temporarily high curvature. In these segments, the microtubule lattice experiences compressive and tensile stresses which—as fracture mechanics teaches^28,29^—at a sufficiently high level cause breaking.

From the perspective of mechanochemistry^16^, the breaking of a strained microtubule is a stochastic event, because it originates in the stochastic breaking of one or more intermolecular bonds. Rather than being characterized by a specific curvature which separates a stable from a breaking regime, we expect a distribution of observed breaking curvatures arising from a common stochastic process. The challenge is thus to determine the distribution of breaking curvatures and properly normalize it with the observed number of microtubule segments of that curvature to determine the curvature-dependent probability of breaking in the given time interval.

### Measuring gliding and breaking curvature distributions

The curvature of gliding and breaking microtubules was determined using the three-point method, which according to Bicek et al. accurately reproduced the curvature distribution^30^. In this method, the curvature is estimated by dividing the change in angle along a segment of a microtubule by the segment length. We first generate a highly resolved estimate of the microtubule shape (in a procedure slightly different from those proposed by Xiao et al.^31^) by interpolating the microscopy images with a pixel size of 65 nm eightfold down to a pixel size of 8.1 nm, identifying the contour of the microtubules by thresholding, and estimating the microtubule center position by skeletonizing (see “Materials and methods”). Only microtubules with a length of at least 3 μm were considered, because shorter microtubules yielded poor estimates of their shape. The pixel positions of the skeletonized microtubule yield a noisy estimate of the microtubule shape. The shape is translated into a sequence of segments by defining a series of points starting from a tip of the microtubule separated by a specific path length. For small segment lengths, the localization noise is a dominant contributor to the angle change, whereas for large microtubule segment lengths the three-point method can underestimate high curvature events.

**Figure 2:**
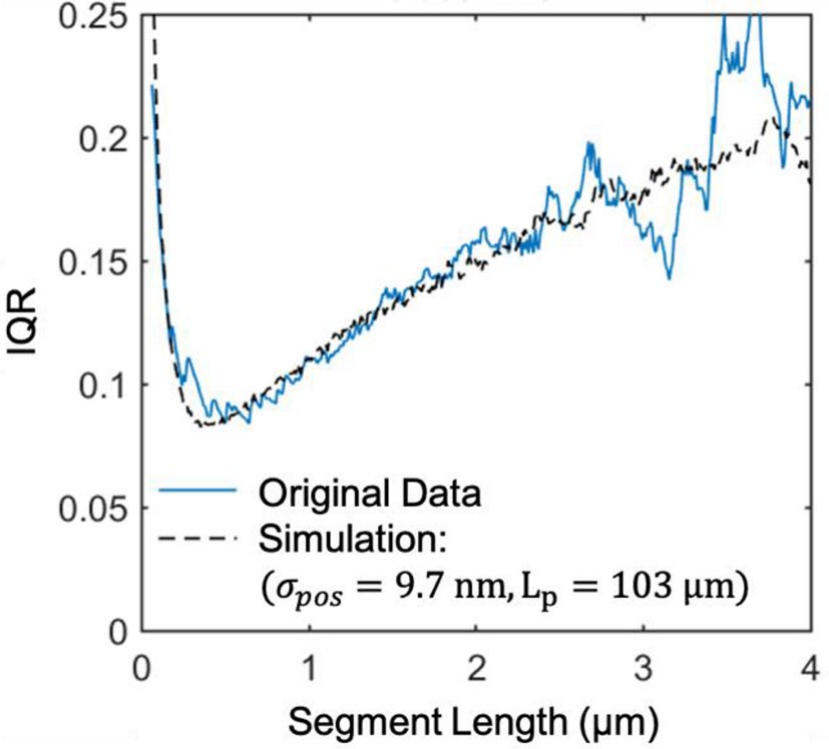
Interquartile range for the distribution of angle changes as a function of segment length. Experimental data for a kinesin surface density and ATP concentration of 500 μm^−2^ and 1 mM (blue, solid line). Simulation of a microtubule undergoing a persistent random walk with persistence length $L_{P} = 103$ μm corrupted with uniformly distributed noise on $\left[ { - \sigma_{pos} , \sigma_{pos} } \right]$, with $\sigma_{pos} = 9.7\;{\text{nm}}$ (black, dashed line).

To calculate the curvature distribution, we chose a segment length of 0.5 μm, which minimizes the interquartile range of the distribution of change in angle (Fig. 2; see SI Sect. 1). This choice matches the choice Odde et al.^13^ made to analyze in vivo microtubule bending and breaking and can be justified by the following theoretical argument: According to the persistent random walk model for a microtubule, the distribution of the instantaneous change in angle is zero-mean normal with standard deviation of $\sqrt {\Delta s/L_{P} }$, where Δs is the segment length and *L*_*P*_ is the persistence length. At the same time, there is noise in the measurement of the position of a microtubule segment, which corrupts the measurement of the angle change by a magnitude of $\sin \left( {\Delta \theta } \right) \approx \Delta \theta = 2\sigma_{pos} /\Delta s$, where $\sigma_{pos}$ is the error in the measurement of the position, and the factor of two comes from taking the difference of two points to attain the vector of the orientation. Considering that the trajectory of a kinesin-propelled microtubule has a persistence length on the order of 0.1 mm^20^, and our super-resolving procedure has 10 nm accuracy, we expect the signal and noise contributions to the angle measurement to be equal when $\sqrt {\Delta s/L_{P} } = 2\sigma_{pos} /\Delta s$, or $\Delta s = 400\;{\text{nm}}$, consistent with the data. A simulation of a persistent random walk with persistence length ${L}_{P}=$ 103 μm corrupted with ${\sigma }_{pos}=$ 9.7 nm uniformly distributed noise gives a match with the experimental data (Fig. 2, black dashed line, Supplementary Information Sect. 1). All microtubules exhibiting at least one segment with a curvature greater than 0.4 μm^−1^ were manually examined to confirm that the high curvature was not due to an error in the automated analysis of the microscopy images.

**Figure 3:**
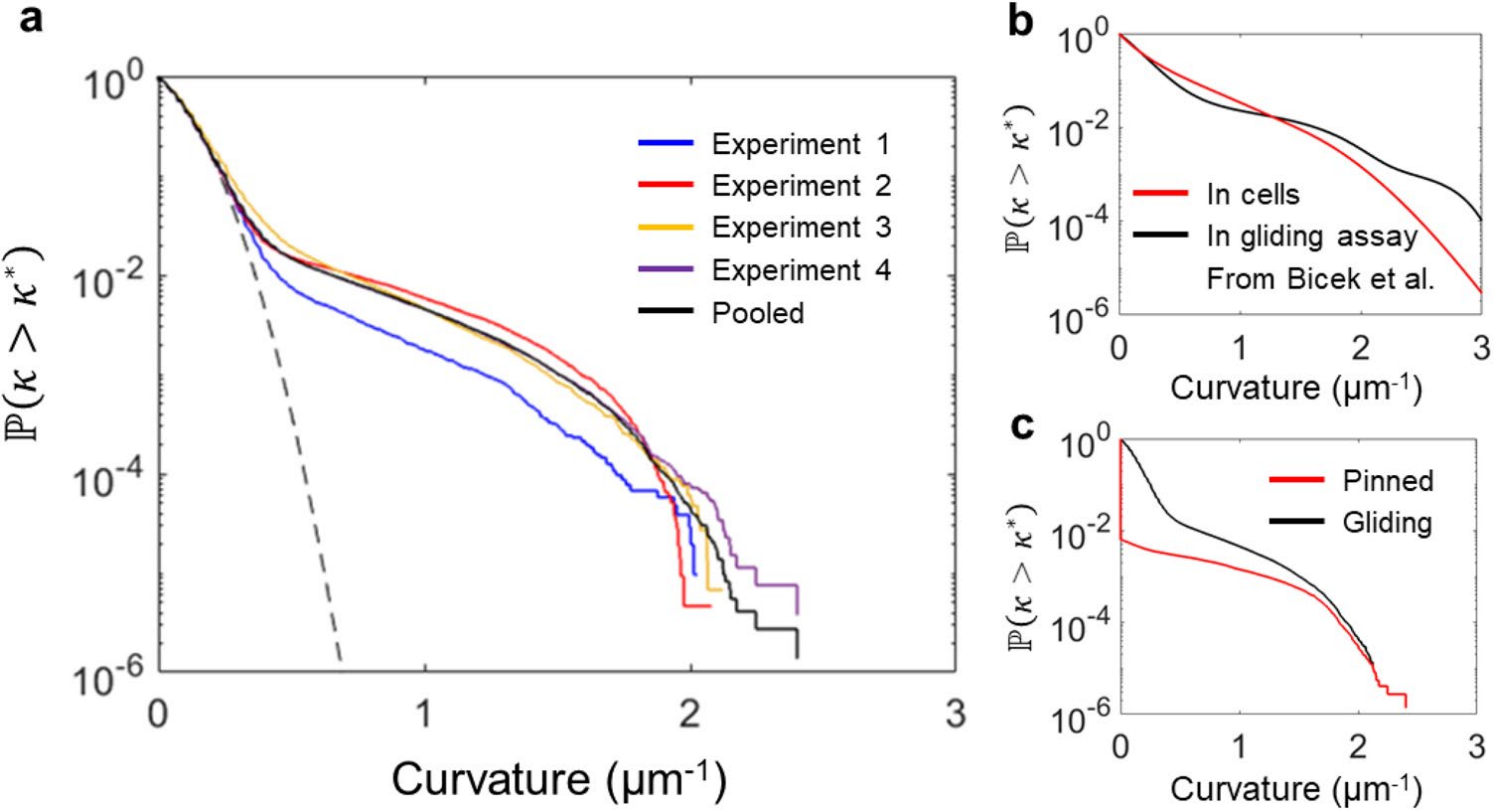
Curvatures of all microtubule segments. (**a**) The complement of the cumulative distribution function of the curvatures of 0.5 μm microtubule segments for repeated experiments with 1 mM ATP, 500 μm^−2^ kinesin (Experiment 1: N = 107,107–blue; Experiment 2: N = 219,733–yellow; Experiment 3: N = 151,324–red, Experiment 4: N = 269,897–purple). The black solid line is the cumulative distribution of pooled data from the four individual experiments. The dashed black line indicates the expected curvature distribution for a persistent random walk with a 100 μm persistence length. (**b**) Curvature distributions found by Bicek et al.^33^ in motility assays (black) and living cells (red). (**c**) The segments of the pinned microtubules (red line) represent a fraction of all segments (black line, same as in **a**) which increases with curvature. The curvature distribution of only pinned microtubule segments is zero-padded to match the population size of all microtubule segments.

The curvature distribution of all observed microtubule segments is plotted as the complement of the cumulative distribution function in Fig. 3a, because it permits the plotting on a logarithmic scale. Although a half-Gaussian distribution is expected for the curvature probability distribution of a persistent random walk (black dashed line in Fig. 3)^30^, and spline-smoothed trajectories of actin filaments gliding on surface-bound myosin were found to exhibit an exponential distribution of curvatures^32^, we find a more complex, multi-regime shape. For curvatures below 0.3 μm^−1^, the observed distribution matches the expected half-Gaussian distribution of a persistent random walk with a persistence length of 100 μm^20^. For curvatures above 0.3 μm^−1^, an exponential tail is observed. The fraction of total segments that fall into this exponential tail varies between experimental replicates, but the slope of the exponential tail remains the same. The mean curvature of the analyzed microtubules segments is 0.12 μm^−1^, which is significantly lower than the mean curvature of 0.39 μm^−1^ of the microtubules in cells studied by Odde et al.^13^ Our curvature distribution is in excellent agreement with the curvature distribution of microtubules gliding on surface-adhered kinesins in vitro presented by Bicek et al.^33^, which is shown together with their in vivo curvature distribution (Fig. 3b).

Our manual analysis of the curvature of the breaking microtubule segment prior to each breaking event revealed that most breaking events occur after a microtubule is pinned (most likely to an inactive kinesin)^19^, with only 5 unpinned breaking events out of 116 total events. However, we observe many gliding microtubules which are not pinned and do not break but exhibit similarly high curvatures as pinned microtubules. Only 556 of the 4461 microtubules having a segment with a curvature greater than 0.5 μm^−1^ are pinned (a microtubule observed over several frames is counted repeatedly here). This indicates that—in our experimental setup—high curvature alone rarely leads to breaking, whereas pinning combined with high curvature leads to frequent breaks. A pinned microtubule experiences additional stresses (as discussed below) that increase the breaking rate.

The smoothly gliding microtubules exhibit 5 breaking events in 743,123 observed segments, each representing a time interval of 2 s, which yields an overall breaking rate of 0.4 ± 0.1 mm^−1^ min^−1^. This rate is six-fold lower than the overall breaking rate of 2.3 mm^−1^ min^−1^ for microtubules in cells determined by Odde et al.^13^ and precludes an analysis of the curvature dependence due to the small number of breaking events.

**Figure 4:**
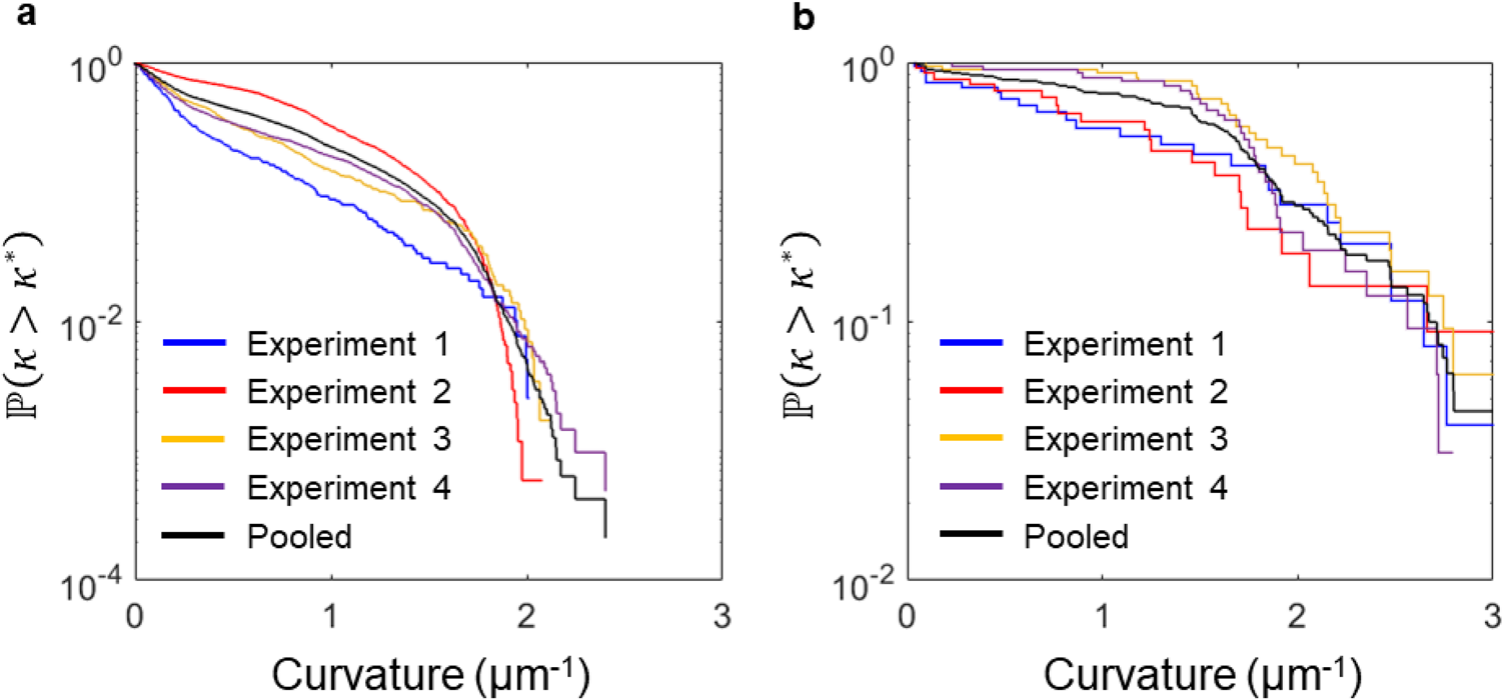
Distributions of curvatures of pinned microtubule segments and of breaking events. (**a**) The complement of the cumulative distribution function of the curvatures of 0.5 μm microtubule segments is shown for repeated experiments with 1 mM ATP, 500 μm^−2^ kinesin (Experiment 1: N = 411–blue; Experiment 2: N = 1742–yellow; Experiment 3: N = 620–red, Experiment 4: N = 2165–purple). The black solid line is the cumulative distribution of pooled data from the four individual experiments. (**b**) The complement of the cumulative distribution function of the curvatures of 0.5 μm microtubule segments breaking in the next frame is shown for repeated experiments with 1 mM ATP, 500 μm^−2^ kinesin (Experiment 1: N = 25–blue; Experiment 2: N = 22–yellow; Experiment 3: N = 32–red, Experiment 4: N = 32–purple).

The pinned microtubules exhibit 111 breaking events in 4938 observed segments, yielding an overall breaking rate of 1300 ± 400 mm^−1^ min^−1^. This 3000-fold increase shows that pinning creates a categorically different situation, which we will investigate now separately from the smoothly gliding microtubules (Fig. 4). The complement of the cumulative curvature distribution of the segments of the pinned microtubules (Fig. 4a) mirrors the shape of the tail of the curvature distribution of all microtubule segments (Fig. 3), even though the pinned microtubule segments (N = 4938) contribute only a small fraction of the observations constituting the tail (N ≈ 41,000). The segments of pinned microtubules have an average curvature of 0.59 μm^−1^. The curvature of the segments breaking in the next frame is approximately uniformly distributed over the observable range of curvatures (0–3 μm^−1^). The data shown in Fig. 4 form the foundation for the analysis of the curvature dependence of the breaking rates of pinned microtubules.

### Curvature-dependent breaking rates

In order to estimate the curvature dependent breaking rate of pinned microtubules, we construct a minimal non-parametric model with the single assumption that the breaking process is memoryless; i.e., breaking is only dependent on the curvature experienced by the breaking point immediately before the break, and does not account for any fatigue or defects that a microtubule may have accumulated throughout its history of gliding across the surface. This assumption is justified if a single intermolecular rupture event initiates a fracture which rapidly grows and ends in breaking, so that the initial rupture event is the rate-determining step for breaking as discussed by Zhurkov for polymeric materials^34^. A memoryless process has a constant hazard rate, and therefore an exponential distribution of event times for a given curvature. As a result, with ${\mathbb{P}}\left( {break{|}\kappa } \right)$ denoting the probability of a break occurring in a segment of length *Δs* within an interval of time *Δt*, we can write:

1 $$\begin{array}{*{20}c} {{\mathbb{P}}\left( {break{|}\kappa } \right) = 1 - \exp \left( { - \lambda \left( \kappa \right)\Delta s{\Delta }t} \right)} \\ \end{array}$$

where $\lambda \left(\kappa \right)$ is the curvature dependent breaking rate. Using Bayes’ Formula, we can rewrite this quantity in terms of the probability density functions of breaking curvatures, ${\mathbb{P}}\left( {\kappa {|}break} \right)$, and nonbreaking curvatures, ${\mathbb{P}}\left( {\kappa {|}nonbreak} \right)$, and the proportion of total microtubules that did and did not break, ${\mathbb{P}}\left( {break} \right)$ and ${\mathbb{P}}\left( {nonbreak} \right)$, respectively:

2 $$\begin{array}{*{20}c} {{\mathbb{P}}\left( {break{|}\kappa } \right) = \frac{{{\mathbb{P}}\left( {\kappa {|}break} \right){\mathbb{P}}\left( {break} \right)}}{{{\mathbb{P}}\left( {\kappa {|}nonbreak} \right){\mathbb{P}}\left( {nonbreak} \right) + {\mathbb{P}}\left( {\kappa {|}break} \right){\mathbb{P}}\left( {break} \right)}}} \\ \end{array}$$

By combining Eqs. (1) and (2), the estimate for the curvature dependent breaking rate is obtained as:

3 $$\begin{array}{*{20}c} {\lambda \left( \kappa \right) = - \frac{1}{\Delta s \Delta t} \cdot \ln \left( {\frac{{{\mathbb{P}}\left( {\kappa |nonbreak} \right){\mathbb{P}}\left( {nonbreak} \right)}}{{{\mathbb{P}}\left( {\kappa |nonbreak} \right){\mathbb{P}}\left( {nonbreak} \right) + {\mathbb{P}}\left( {\kappa |break} \right){\mathbb{P}}\left( {break} \right)}}} \right)} \\ \end{array}$$

**Figure 5:**
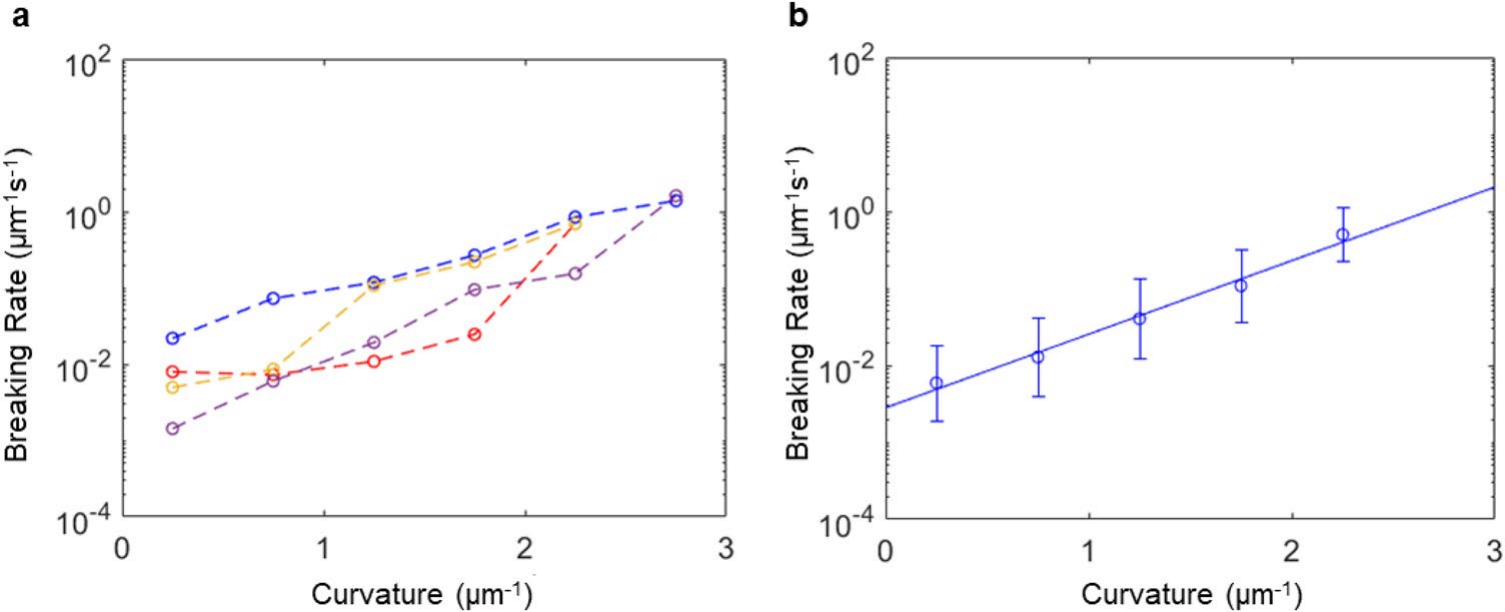
Breaking rate as a function of local curvature. (**a**) The 4 individual experiments with 1 mM ATP, 500 μm^−2^ kinesin. (**b**) The combination of the four experiments. The circles are estimated using the Bayesian formula described in Eq. (3); the solid line is fit using maximum likelihood estimation.

The breaking and nonbreaking probabilities due to curvature are estimated by binning microtubule segments in 6 curvature bins ($0 - 0.{5}, \, 0.{5} - {1}, \ldots ,{ 2}.{5} - {3}$ μm^−1^). These estimates are used to evaluate $\lambda \left(\kappa \right)$ as described in Eq. (3), using $\mathrm{\Delta s}=0.5$ μm (i.e. the segment length) and $\mathrm{\Delta t}=2$ s (i.e. the time between frames). The resulting curvature-dependent microtubule breaking rate, $\lambda \left(\kappa \right)$, with units of 1 μm^−1^ s^−1^ is shown in Fig. 5.

The observed exponential increase with curvature of the microtubule breaking rate is expected, since the stress on a volume element in a bent rod made from an isotropic material increases linearly with curvature, and since according to the Bell equation^35^ a linearly increasing force on an intermolecular bond leads to an exponentially increasing unbinding rate. Therefore, we fit the breaking rate as function of the curvature κ with an exponential function parametrized by the breaking rate at zero curvature ${\lambda }_{0}$ and the characteristic breaking radius ${r}^{*}$:

4 $$\begin{array}{*{20}c} {\lambda \left( \kappa \right) = \lambda_{0} \exp \left( {\kappa r^{*} } \right)} \\ \end{array}$$

The parameters are determined by maximum likelihood estimation of the unbinned data since the number of events varies between bins and the breaking rate depends strongly on the curvature (SI Sect. 3). The fit value across all experiments for the breaking rate at zero curvature ${\lambda }_{0}$ is found to be 1.8 mm^−1^ s^−1^ (1.1–3.0 mm^−1^ s^−1^, 95% confidence interval) and the characteristic breaking radius ${r}^{*}$ is 2.3 μm (2.0–2.7 μm, 95% confidence interval), which corresponds to a characteristic curvature of breaking of 0.43 μm^−1^ (0.38–0.49 μm^−1^, 95% confidence interval).

In order to test if the parameters depend on the microtubule length, we split the population of microtubules into two roughly equally large groups (2691 short and 2247 long segments) based on their length and fitted the breaking rates for the short (L < 6 μm) and long (L ≥ 6 μm) microtubules independently. We found no statistically significant difference between the fit parameters for short and long microtubules. The full analysis of the length dependence is shown in SI Sect. 5.

In order to test if the parameters depend on the time of the break, we split the observations into two roughly equally large groups (2987 early and 1951 late segments) based on their length and fitted the breaking rates for the early (t < 900 s) and late (t ≥ 900 s) observations independently. We found no statistically significant difference in the fit parameters between early and late observations. The full analysis of the time dependence is shown in SI Sect. 6. This justifies the assumptions of the model that the microtubules do not age as a result of smooth gliding and that their breaking rate is independent of their length.

### Breaking rates per dimer length

**Figure 6:**
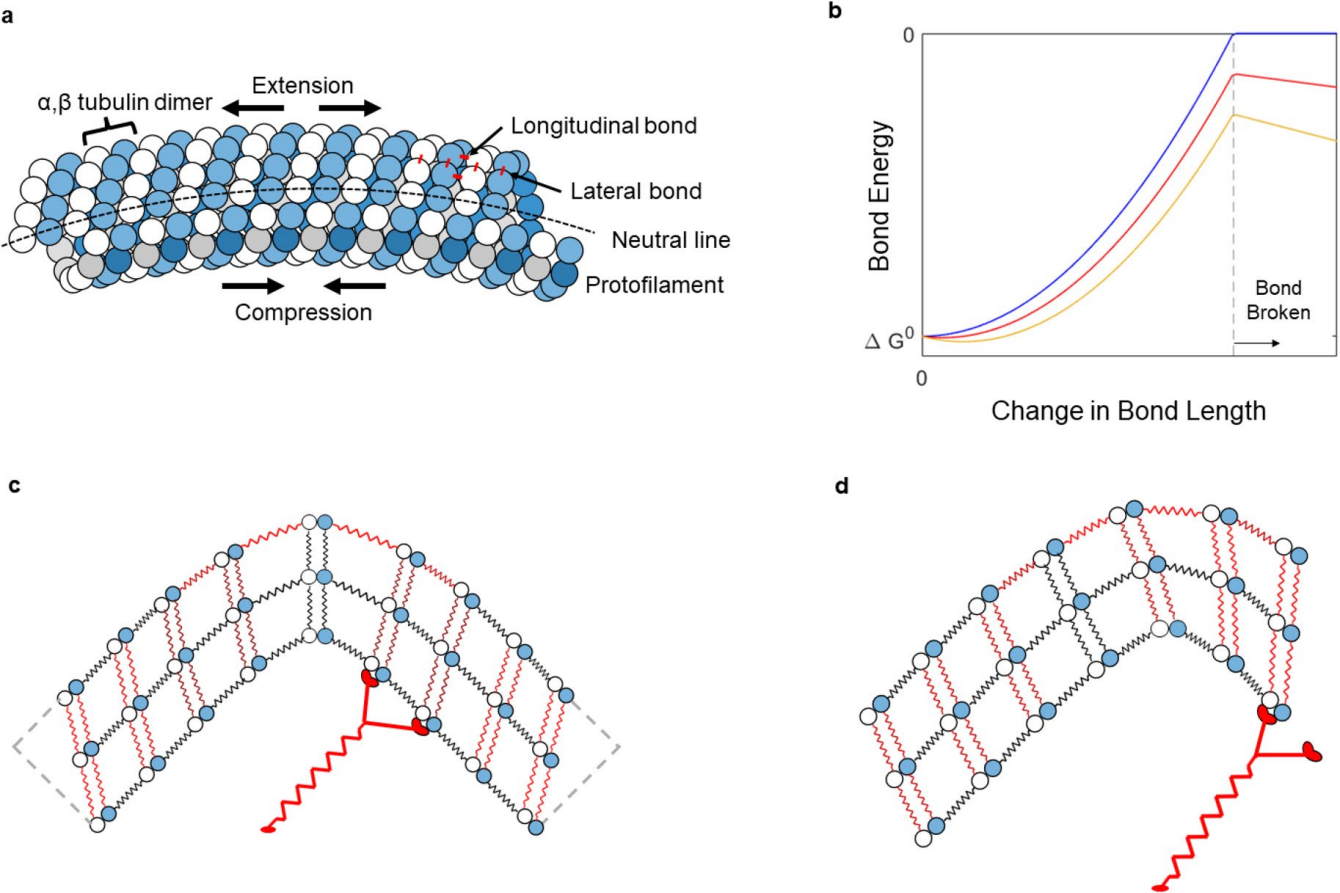
Strain distribution in a curved microtubule. (**a**) Lateral view. (**b**) Bond energy as a function of bond length in the absence of a bending induced stretching force (blue line), and in the presence of two levels of a bending induced stretching force (red and yellow lines). Schematic of bond energies during bending of (**c**) unpinned and (**d**) pinned microtubules. Red shading indicates the presence of tensile force on longitudinal and lateral dimer-dimer bonds.

As a microtubule bends, the neighboring dimers at the outer edge are pulled apart, the neighboring dimers at the center of the microtubule maintain their spacing, and the neighboring dimers at the inner edge are compressed (Fig. 6). In this situation, the most straightforward way to break the microtubule is to rupture the outermost longitudinal tubulin-tubulin dimer bond, which increases the load on the remaining bonds and causes a cascading failure of longitudinal tubulin-tubulin bonds until the microtubule breaks.

The lengthening of the outermost tubulin-tubulin dimer bond, *x*, as a function of the curvature κ is given by:

5 $$\begin{array}{*{20}c} {x = \frac{d}{2}l_{dimer} \kappa } \\ \end{array}$$

where *l*_*dimer*_ = 8 nm is the length of the tubulin dimer, and d = 20 nm is the center-to-center distance of the outermost protofilaments^1^. Thus, at the maximum observed curvatures of 3 μm^−1^ the tubulin-tubulin bond is stretched by 0.2 nm.

The acceleration of the tubulin-tubulin unbinding rate is determined by the reduction in the activation energy $\Delta E$ for the unbinding process according to the Arrhenius equation:

6 $$\begin{array}{*{20}c} {k_{dimer} = A\exp \left( {\frac{\Delta E}{{k_{B} T}}} \right)} \\ \end{array}$$

where A is the unbinding rate in the absence of force, *k*_*B*_ is the Boltzmann constant and T is the temperature.

The stretching of an isolated bond by a defined distance *x* reduces the activation energy by $k{x}^{2}/2$ where *k* is the spring constant of the longitudinal tubulin-tubulin bond. According to Eqs. (5) and (6), this implies that the log-linear plot in Fig. 5b should show a quadratic increase of the logarithm of the breaking rate with curvature, which is not observed. However, the outermost tubulin-tubulin bond is not an isolated bond but should rather be seen as part of a chain of bonds placed under a load, as described by Pampaloni et al.^5^ The extension of a single bond then reduces the strain on the chain only marginally, which maintains the initial force on the bond at a nearly constant level. In this situation, the change in activation energy is given by:

7 $$\begin{array}{*{20}c} {\Delta E = kx^{*} x = kx^{*} \frac{{\text{d}}}{2}l_{dimer} \kappa } \\ \end{array}$$

where x* is the distance to the transition state. In combination with Eq. (5) this yields the observed linear dependence of the logarithm of the unbinding rate on the curvature.

The unbinding rate of the outermost tubulin dimers in the absence of force, A, is found to be 1.5⨯10^–5^ s^−1^ (9⨯10^–6^–2.4⨯10^–5^ s^−1^, 95% confidence interval) by multiplying the breaking rate at zero curvature ${\lambda }_{0}$ by the length of a tubulin dimer (8 nm), since the rupture of an outermost tubulin-tubulin bond is assumed to be solely responsible for the initiation of breaking. The characteristic breaking radius r* yields the distance to the transition state by combining Eqs. (4–7):

8 $$\begin{array}{*{20}c} {x^{*} = \frac{{2k_{B} Tr^{*} }}{{kdl_{dimer} }}} \\ \end{array}$$

Using a longitudinal spring constant of 0.7 N/m as described by VanBuren et al.^36^, our measurement of the characteristic breaking radius implies a value of x* = 0.2 nm.

## Discussion

The experimental observations can be interpreted in the context of the mechanochemical model of microtubule structure developed by VanBuren, Cassimeris and Odde^36^. The model posits that each tubulin dimer is connected to adjacent tubulins via two longitudinal bonds with a bond energy of − 9.4 k_B_T and a spring constant of 0.7 N/m and four lateral bonds with a bond energy of − 3.2 k_B_T and a spring constant of 0.4 N/m (Fig. 6d). The model also accounts for the curling of tubulin dimers as a result of GTP hydrolysis, however for our paclitaxel-stabilized microtubules curling is not present^36^. The attempt frequency for the removal of a tubulin dimer from the lattice is chosen as 2⨯10^6^ s^−1^. Strain in the tubulin lattice increases the bond energies (from their negative values) according to a parabolic potential until a zero value is reached, at which point the bond breaks and the interaction ceases. According to this model, a tubulin dimer with all bonds intact and unstrained will leave the lattice at a rate of 4⨯10^–8^ s^−1^. Breaking the two lateral bonds on one side will increase the energy by 2 × 3.2 k_B_T and increase the rate at which the dimer leaves the (unstrained) lattice 600-fold to 2⨯10^–5^ s^−1^. Breaking a longitudinal bond on one end will increase the energy by 9.4 k_B_T and the rate at which the dimer leaves 12,000-fold. The application of increasing force to lateral and longitudinal bonds will gradually increase the unbinding rate until the bonds are broken, increasing the unbinding rate up to 600-fold for lateral bonds under strain and up to 12,000-fold for longitudinal bonds under stress from the 4⨯10^–8^ s^−1^ baseline rate.

The experimental results can be interpreted using the model: (1) The smoothly gliding microtubules break at a rate of 0.4 ± 0.1 mm^−1^ min^−1^, which corresponds to a rate of (5 ± 2)⨯10^–8^ s^−1^. The equality between the breaking rate of the freely gliding microtubules and the predicted unbinding rate of the outermost tubulin (4⨯10^–8^ s^−1^) suggests that the removal of the outermost tubulin from the lattice—and not just the breaking of a longitudinal bond as discussed above—initiates the breaking. (2) An increasing curvature exponentially increases the breaking rate 250-fold as the curvature increases from zero to 2.5 μm^−1^. This behavior aligns with VanBuren et al.’s model, specifically Eqs. (12, 13), of strain-facilitated unbinding if we assume that longitudinal strain is distributed along a protofilament as described above. The distance to the transition state for the breaking of longitudinal bonds implied by our measurements of x* = 0.2 nm is close to the distance to longitudinal bond rupture of 0.3 nm implied by VanBuren et al.’s potential energy surface for the longitudinal bond (Fig. 6b). (3) The main puzzle is why the pinned microtubules break at a 400-fold higher rate than smoothly gliding microtubules (λ(κ= 0) = 1.5*10^–5^ s^−1^ vs. λ(κ = 0) = 5.4*10^–8^ s^−1^). Our explanation for this is that the peculiar geometry of a pinned microtubule places stress not only on the longitudinal bonds but also on the lateral bonds near the tip of the microtubule and thereby adds to the destabilization of the tubulin lattice.

This stress on the lateral bonds originates from the sliding of the protofilaments relative to each other as they bend as described by Pampaloni et al. and others^5^. The sliding is resisted by the lateral bonds, is most pronounced towards the ends of the microtubule, and zero in the center of the microtubule where the combined forces are balanced (which can be far away from the point of highest curvature). The 2 nm difference in bending radius between the outermost protofilaments and its adjacent protofilaments causes sliding (up to 0.2 nm for a 90 degree turn), and the sliding is largest towards the end of the microtubule (Fig. 6c, d). This means lateral bonds are largely unstrained when the curved segment is in the center of the microtubule (as is mostly the case in a smoothly gliding microtubule), but lateral and longitudinal bonds are strained near the tip of a pinned microtubule. We do not attempt to quantitatively model this process beyond noting that only a modest stress on the lateral bonds is required, since the employed stabilizing agent paclitaxel (taxol) may modulate the inter-protofilament interactions and thereby introduce large uncertainties in the parameter values^37^. The details of the mechanics of the breaking events may become accessible to state-of-the-art simulation approaches^38^. This explanation is also consistent with the remark in Odde et al.^13^ stating: “buckling microtubules to radii of curvatures < 1 μm by using optical tweezers does not result in breaking, even if high curvature is maintained for up to one hour”, since a buckled microtubule also has a geometry where the stresses on longitudinal and lateral bonds is not co-located. Alternative explanations for the difference in the breaking rate between smoothly gliding and pinned microtubules, including the role of compressive forces, torque and forced kinesin unbinding, were considered and rejected by us (see SI Sect. 7).

A comparison between our observations and Odde et al.’s earlier observations of breaking microtubules in cells is fascinating. Odde et al.^13^ observe an exponential distribution of microtubule curvatures with a mean curvature of 0.4 μm (5 s intervals, 0.5 μm segments, N = 251,412) and a uniform distribution of breaking events as a function of curvature (n = 24). This—in good agreement with Eq. (4)—implies an exponential increase in the breaking rate with curvature with a breaking rate at zero curvature ${\lambda }_{0}$ of 5*10^–6^ μm^−1^ s^−1^ (0.3 mm^−1^ min^−1^ or 4*10^–8^ s^−1^ per dimer length) and a characteristic curvature of breaking of 0.4 μm^−1^. The equality between the characteristic curvatures of breaking determined from our observations and Odde et al.’s is pleasing and suggests that the same intermolecular unbinding event (with the same distance to the transition state) drives breaking in both situations. Note that Odde et al.’s breaking rate at zero curvature ${\lambda }_{0}$ is about ten-fold lower than their average breaking rate because curved segments account for most breaking events, and that the breaking rate at zero curvature ${\lambda }_{0}$ is identical to the unbinding rate of a tubulin dimer from the lattice according to VanBuren et al.’s theory. Odde et al. image stationary microtubules which largely maintain their shape over time. In this situation, one would expect increased breaking if an extended exposure to high curvatures accelerates breaking, yet the breaking rates are similar to those of our smoothly gliding microtubules and match the theoretical dimer unbinding rate calculated without considering potential bond reforming.

The picture of the breaking events which emerges is that the removal of a tubulin dimer from the lattice initiates breaking, that curvature strains the longitudinal bonds and accelerates breaking according to a “constant force” Bell equation, that pinning of kinesin-propelled microtubules accelerates breaking 250-fold by causing strain of the lateral tubulin-tubulin bonds in the highly curved segments near the microtubule tip, and that the experimental observations are consistent with VanBuren et al.’s mechanochemical model of the microtubule.

A fascinating corollary of these insights regarding the curvature-dependence of microtubule breaking is that they together with a curvature-increasing process originating from a compression of segments (as a consequence of motor forces) predict a stable curvature distribution which is identical to the experimentally observed one for pinned microtubules in Fig. 4a (see SI Sect. 8). This corollary emerges from equating the process for removing microtubule segments of a specific curvature from the distribution (breaking) with a process of continuous increase of microtubule segment curvature due to motor forces (or anterograde flow in cells^33,39^).

This suggests—as described previously by Bicek et al.^33^—that in our gliding assay, smoothly gliding microtubules have a curvature distribution determined by the random fluctuations of the advancing microtubule tip, while pinning events lead to a build-up of high curvature segments subject to breaking. In cells, anterograde flow locally buckles microtubule segments, yielding a similar curvature distribution^39^.

While Odde et al. discusses the potential role of severing enzymes in the in vivo breaking events^13^, these enzymes are absent in our in vitro experiments, yet we obtain substantially identical results. This suggests that severing enzymes are operating under biological control in addition to the mechanochemical breaking mechanism^40^, and that curvature-induced breaking affects the microtubule length distribution in cells^41^. Waterman-Storer and Salmon stated: “we suspect that mechanics are relevant, although breaking MTs by bending in vitro has not been documented. However, this does not rule out the activity of severing proteins”^12^. Our work now shows that mechanics can take full responsibility for breaking of bending microtubules, given that our average curvature for breaking microtubules of (1.7 ± 1.2 μm^−1^) (Fig. 4b) corresponds to their average radius of curvature for breaking microtubules (0.6 ± 0.15 μm). Nevertheless, biological structures and situations are diverse and complex and are not fully reproduced by in vitro assays or even fibroblasts. Microtubule-associated proteins (MAPs) and other factors alter microtubule stability, and their impact can vary across cell types and change during the lifetime of an individual cell^42^. For example, axons exhibit highly contorted microtubules at branch points whose stability is controlled by MAP7^43^. Additional exploration of the mechanobiology of microtubules is therefore warranted.

Interestingly, we do not see evidence of cross-sectional flattening of bending microtubules or microtubule degradation due to kinesin motor activity. Memet et al. bent GMPCPP-stabilized microtubules with optical tweezers and identified the onset of cross-sectional buckling (kinking) at a curvature of only 0.2 μm^−1^, after which the rigidity of the microtubule was greatly reduced^6^. Our curvature distribution does not show a deviation from the expected Gaussian until 0.5 μm^−1^, possibly because paclitaxel stabilization alters the microtubule mechanics^6^. Triclin et al. showed that kinesin translocation along microtubules can induce defects which can be repaired by tubulin binding from solution^25^. Since the free tubulin concentration in our experiments is very low, motor activity should lead to accumulating damage and a breaking rate which increases with time, which we do not observe.

## Conclusion

Microtubule breaking is a fascinating process at the intersection of supramolecular chemistry and biomechanics. Building on the prior work by Odde et al.^13^ and Bicek et al.^33^, who established that microtubule breaking in cells is strongly curvature dependent, and that the microtubule curvature distribution in cells and in vitro gliding motility assays is identical, we analyzed the curvature dependence of microtubule breaking in in vitro gliding motility assays. The first new insight is that breaking mainly occurs towards the tip of pinned microtubules, which we explain as a consequence of protofilament sliding in curved microtubules. The second insight is that the breaking rate increases exponentially with curvature, consistent with the mechanochemical model of the microtubule structure of VanBuren, Cassimeris and Odde^36^. This implies that the presence of severing enzymes is not required for microtubule breaking. Finally, we find that the combination of curvature production by longitudinal compression and curvature destruction by breaking yields a steady state curvature distribution which matches the experimental observations. These findings contribute to our understanding of microtubule mechanics and are informing efforts to engineer hybrid nanodevices using gliding microtubules as active components^2,14^ or contractile materials from microtubules and kinesin motors^44^.

## Materials and methods

Microtubules were polymerized from a 20 μg aliquot of rhodamine-labelled, lyophilized tubulin (Cytoskeleton Inc., TL670M) with 6.25 μL polymerization buffer. The polymerization buffer consisted of BRB80 buffer, with 4 mM magnesium chloride (MgCl_2_), 1 mM GTP and 5% dimethylsulfoxide. BRB80 buffer is composed of 80 mM piperazine-N,N′-bis(2-ethanesulphonic acid), 1 mM MgCl_2_ and 1 mM ethylene glycol tetraacetic acid (EGTA), adjusted to pH of 6.89 with potassium hydroxide (KOH). The resulting solution was then incubated on ice for 5 min before being transferred to a 37 °C water bath for 30 min. The microtubules were then diluted a 100-fold into BRB80 buffer and stabilized with 10 μM paclitaxel.

Kinesin-1 from wild-type, full-length Drosophila was expressed by the team of G. Bachand at the Center for Integrated Nanotechnologies (Sandia National Laboratory) in *Escherichia coli* and purified using a Ni–NTA column. The kinesin was then nitrogen frozen in a buffer consisting of 40 mM imidazole, 300 mM NaCl, 0.76 g/L EGTA, 37.2 mg/L EDTA, 50 g/L sucrose, 0.2 mM TCEP, 50 µM Mg-ATP, the buffer being at pH 7. As measured from absorbance at 280 nm the concentration of the kinesin is 3.16 mg/mL. The functional kinesin density was computed from landing rate experiments^22^. The undiluted bulk solution of kinesin would result in our flow cells in a surface density of 11,000 ± 2000 μm^−2^.

Flow cells were assembled from a longer coverslip (60 mm × 25 mm) and a shorter one (22 mm × 22 mm), separated by two strips of double-sided adhesive tape. Before being assembled into flow cells, the coverslips were washed twice with ethanol, twice with ultrapure water, sonicated for 5 min and dried in an oven at 75 °C.

Experimental procedure. A solution of 0.5 mg/mL casein in BRB80 buffer was flowed into a flow cell. After 5 min, the solution was exchanged with the kinesin motor solution (kinesin to coat the surface at 500 ± 100 μm^−2^; 0.5 mg/mL casein; 1 mM ATP), which in turn was exchanged after 5 min with the microtubule solution (16 nM tubulin, 0.5 mg/mL casein, 10 μM paclitaxel; 20 mM D-glucose, 20 μg/mL glucose oxidase, 8 μg/mL catalase, 10 mM dithiothreitol and 0.01 or 1 mM ATP in BRB80). After another 5 min, the microtubule solution was exchanged with an enzymatic antifade solution (0.5 mg/mL casein, 10 μM paclitaxel; 20 mM D-glucose, 20 μg/mL glucose oxidase, 8 μg/mL catalase, 10 mM dithiothreitol and 0.01 or 1 mM ATP in BRB80)^45,46^ in order to remove unbound microtubules from the solution. All experiments were performed at 24 ± 1 °C.

Image acquisition and data analysis. The flow cells were imaged using a Nikon Ti-E epi-fluorescence microscope equipped with an iXON DU897 Ultra electron-multiplying charge-coupled device (EMCCD) camera (Andor) and a 100 × oil objective (NA = 1.45). For each flow cell, a field of view was randomly selected and images were taken every 2 s for 30 min. The exposure time was 200 ms for all images.

Selection of Pinned Microtubules. Each pinning event was manually selected based on the movement of the microtubule in between frames. If the tip of the microtubule is stationary in multiple frames while the body of the microtubules moves, then it is counted as a pinning event. However, there are some microtubules that are pinned for only a single frame and are difficult to identify. In this case, a microtubule is counted as a pinning event if the body of the microtubule buckles and deviates from its original trajectory which is indicative of a pinning event. Examples of pinned microtubules are shown in the supplementary information.

Super-Resolution Microtubule Coordinates. Each recording was filtered using a gaussian filter with a σ of 4 to remove influence of noise on final coordinates. The movies are then upsampled by a factor of 8 using the MATLAB bicubic interpolation function. The interpolated results were thresholded using 4σ above the background intensity as our threshold. The resulting image masks were skeletonized to get the final coordinates used for curvature calculations.

Curvature calculation. Super-resolved microtubule trajectories were decimated to ds = 0.5 μm intervals and the curvature was calculated by measuring the change in angle, dθ between consecutive segments and dividing by the distance between consecutive points: dθ/ds. Breaking curvatures were calculated by manually selecting the coordinate along the microtubule where it breaks. The 0.5 μm segments on each side of the breaking point were used to calculate the breaking curvature.

Empirical distribution functions were calculated using the ‘ecdf’ function of MATLAB.

## Supplementary Information

Supplementary Information 1.

Supplementary Information 2.

Supplementary Information 3.

Supplementary Video 1.

Supplementary Video 2.

Supplementary Video 3.

Supplementary Video 4.

## Data Availability

All data generated during this study are included in this published article. Experimental videos are made available as Supplementary videos S1–4.
