# Supplementary material for: The rate of microtubule breaking increases exponentially with curvature
Source: Sci Rep. 2022 Dec 3;12:20899. doi: 10.1038/s41598-022-24912-0 (PMC9719553; doi:10.1038/s41598-022-24912-0)
Supplement: Supplementary file 1 — Supplementary Information 1. [file 41598_2022_24912_MOESM1_ESM.pdf]

Supplementary Information (SI) to accompany

The rate of microtubule breaking increases exponentially with curvature

Stanislav Tsitkov[1†], Juan B. Rodriguez III[1†], Neda M. Bassir Kazeruni[1†], May Sweet[2], Takahiro Nitta[2] and Henry Hess[1*]

[1] Columbia University, 351L Engineering Terrace, MC 8904

1210 Amsterdam Avenue, New York, NY 10027

2 Applied Physics Course, Faculty of Engineering, Gifu University, Gifu, 503-1193, Japan

† These researchers contributed equally.

Fax: (212) 854-8725

Tel: (212) 854-7749

E-mail: hh2374@columbia.edu

This supplementary information contains eight parts:

1. **Simulating microtubule trajectories and generating Figure 2**
2. **Selection of pinned microtubules**
3. **Maximum likelihood estimation of breaking rate**
4. **Length dependence of microtubule breaking rate**
5. **Time dependence of microtubule breaking rate**
6. **Location and Timing of Breaking events**
7. **Alternative explanations for the difference in the breaking rate between smoothly gliding and pinned microtubules: Compression, kinesin interactions, aging**
8. **Equilibrium Curvature Distribution Due to Breaking**

(1) Simulating microtubule trajectories and generating Figure 2

Microtubule trajectories were simulated according to the persistent random walk model.[1] Each point along the trajectory was described by three numbers: the x position, X, the y position, Y, and the direction of movement relative to the x-axis, θ. In a timestep Δt, the position of the tip of a microtubule gliding at velocity v with persistence length L_P was updated according to:

$$X_{t+\Delta \mathrm{t}} = X_t + v\Delta t \cdot \cos(\theta_t) \tag{1}$$

$$Y_{t+\Delta \mathrm{t}} = Y_t + v\Delta t \cdot \sin(\theta_t) \tag{2}$$

$$\theta_{t+\Delta \mathrm{t}} = \theta_t + Z_t \tag{3}$$

$$Z_t \sim Norm\left(0, \frac{v\Delta t}{L_p}\right) \tag{4}$$

Where Norm(0,V) denotes a normal random variable with mean 0 and variance V. 100 microtubule trajectories of length 1 mm were generated according to the above equations using a discretization of $v\Delta t = \Delta s_{sim} = 1$ nm. To model the effect of discrete pixels, the generated sequence of (X,Y) values was then corrupted by noise which is uniformly distributed on $U \in [-\sigma_{pos}, \sigma_{pos}]$:

$$X_{i,corrupted} = X_i + U_{X,i} \tag{5}$$

$$Y_{i,corrupted} = Y_i + U_{Y,i} \tag{6}$$

$$U_{X,i} \sim Unif\left[-\sigma_{pos}, \sigma_{pos}\right] \tag{7}$$

$$U_{Y,i} \sim Unif\left[-\sigma_{pos}, \sigma_{pos}\right] \tag{8}$$

The angle change distributions for different segment lengths used to calculate the interquartile range in Figure 2 of the main text were generated by the following procedure:

First, we generated a set of 400 segment lengths, linearly spaced between 10 nm and 4 μm. For each segment length, Δs, the corrupted MT trajectories defined by

$$\{(X_{1,c}, Y_{1,c}), \ldots, (X_{n,c}, Y_{n,c})\} \tag{9}$$

(where we have abbreviated 'corrupted' to 'c' for brevity), are subsampled to generate decimated trajectories:

$$\{(X_{i_1,c}, Y_{i_1,c}), \ldots, (X_{i_m,c}, Y_{i_m,c})\} \tag{10}$$

where m is the number of points in the decimated trajectory and the indices i_k or chosen iteratively such that:

$$i_1 = \arg\min_{\substack{\mathrm{k} \\ \mathrm{k}>0}} \|\mathbf{x}_k - \mathbf{x}_0\|_2 \, : s.t. \|\mathbf{x}_k - \mathbf{x}_0\|_2 > \Delta s \tag{11}$$

$$i_2 = \arg\min_{\substack{\mathrm{k} \\ \mathrm{k}>i_1}} \left\|\mathbf{x}_k - \mathbf{x}_{i_1}\right\|_2 \, : s.t. \left\|\mathbf{x}_k - \mathbf{x}_{i_1}\right\|_2 > \Delta s \tag{11a}$$

$$\vdots$$

$$i_{l+1} = \arg\min_{\substack{\mathrm{k} \\ \mathrm{k}>i_l}} \left\|\mathbf{x}_k - \mathbf{x}_{i_l}\right\|_2 \, : s.t. \left\|\mathbf{x}_k - \mathbf{x}_{i_l}\right\|_2 > \Delta s, \tag{11b}$$

where $\mathbf{x}_k$ denotes the point $(X_{k,c}, Y_{k,c})$. In summary, the distance between consecutive points in the decimated trajectory is at least Δs.

The distribution of the change in angle between consecutive points along the decimated trajectory was estimated using the three-point method. In the three-point method, the change in angle, $\Delta\theta_i$, between points $\mathbf{x}_{i-1}, \mathbf{x}_i$, and $\mathbf{x}_{i+1}$ is estimated by:

$$\Delta\theta_i = \arccos \frac{(\mathbf{x}_{i+1} - \mathbf{x}_i) \cdot (\mathbf{x}_i - \mathbf{x}_{i-1})}{\|\mathbf{x}_{i+1} - \mathbf{x}_i\|_2 \|\mathbf{x}_i - \mathbf{x}_{i-1}\|_2} \tag{12}$$

The estimated curvature (κ) is given by: $\kappa = \Delta s / \Delta\theta$.

To obtain the 'Simulation' curve (dashed line) in Figure 2 of the main text, the sets of $\Delta\theta_i|_{i=2}^{m-1}$ from each of the 100 corrupted MT trajectories were pooled together. The interquartile range (IQR) of the pooled set was recorded and associated with the value of Δs that was used to generate the decimated MT trajectory. The entire process (trajectory decimation, angle change calculation, pooling) was repeated for all 400 values of Δs and the resulting IQR estimates were plotted against the values of Δs in Figure 2 of the main text.

For the 'Original Data' curve in Figure 2 of the main text, the same procedure was repeated as for the simulation, only with simulated trajectories being replaced with experimental data.

(2) Selection of pinned microtubules

For the final breaking rate calculation, we manually selected all microtubules in the field-of-view that were pinned to inactive kinesin. A pinning event is identified if the tip of the microtubule is stationary in multiple frames while the body of the microtubules moves. We notice two types of pinning events: spiraling microtubules where the tip is able to rotate and fishtailing microtubules that are unable to rotate. However, there are some microtubules that become pinned and break free again within the time between frames. When this occurs, we can see the microtubule suddenly bend away from its original trajectory. The sharp bend due to pinning is used to identify these brief pinning events that occur in-between frames. Examples of all three types of pinning events are shown in Supplementary Figure 1.

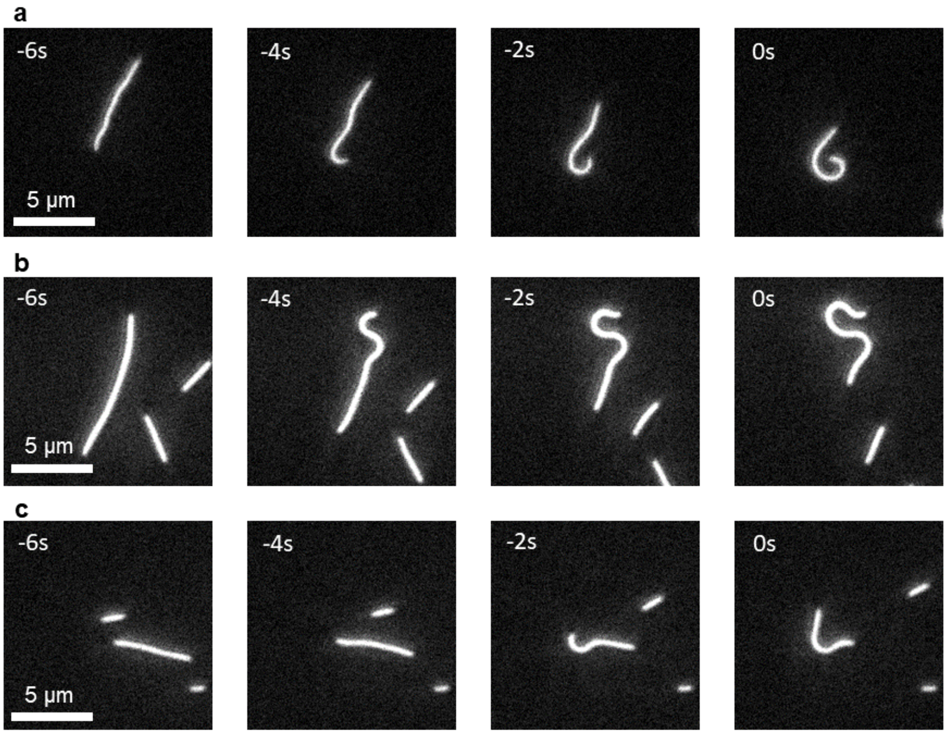

Supplementary Figure 1. Examples of pinned microtubules. (a) Spiraling microtubule; (b) Fishtailing Microtubule; (c) Pinning in-between frames.

(3) Maximum likelihood estimation of breaking rate

The parameters for the breaking rate equation were fit using maximum likelihood estimation of the breaking probabilities of the observed microtubules. The breaking probability as a function of curvature can be written as:

$$\mathbb{P}(break|\kappa) = 1 - exp\big(-exp(a\kappa + b)\big) \tag{1}$$

Where:

$$a = k_{unbinding}; b = ln(\lambda_0 \Delta s \Delta t) \tag{2}$$

The likelihood is the product of the probabilities of all breaking and non-breaking events:

$$L(a,b) = \prod_{k_i \epsilon breaking} P(T_{break} < \Delta t|\kappa_i) \prod_{k_j \epsilon not\ breaking} P(T_{break} > \Delta t|\kappa_j) \tag{3}$$

By taking the natural log of Equation 3 and inserting Equations 1-2, the log-likelihood is obtained:

$$LL(a,b) = \sum_{k_i \epsilon breaking} ln(1 - exp(exp(a\kappa_i + b))) \sum_{k_j \epsilon not\ breaking} exp(a\kappa_j + b) \tag{4}$$

Equation 4 was maximized to find the best fit for parameters a and b. Asymmetric confidence intervals (95%) were constructed by evaluating likelihood profiles for each parameter. The fitted parameters for each individual experiment and for the pooled data are listed in Supplementary Table 1.

Supplementary Table 1. Fitted parameters of the breaking rate for individual experiments & pooled data.

Experiment Number	a = r^* (μm)	b = ln($\lambda_0 \Delta s \Delta t$)
1	1.71 (1.25 to 2.19)	-4.15 (-4.93 to -3.51)
2	1.46 (0.82 to 2.18)	-5.89 (-6.91 to -5.05)
3	2.76 (2.14 to 3.54)	-6.20 (-7.60 to -5.12)
4	2.72 (2.18 to 3.30)	-7.19 (-8.27 to -6.25)
Combined	2.34 (2.04 to 2.66)	-6.30 (-6.84 to -5.80)

(4) Length dependence of breaking rate

To test if the parameters depend on the microtubule length, we split the population of microtubules into two equally large groups based on their length with one population containing all microtubule segments from microtubules shorter than 6 μm and the other containing all microtubule segment from microtubules longer than 6 μm. The same maximum likelihood estimation procedure described in Supplementary Information Section 3 was applied to each population independently to obtain the fit parameters. Part of the MLE procedure includes evaluating the likelihood profile to find the error bounds of the fitted parameters. The parameters and their error are shown in Supplementary Table 2. The calculated parameters for each population are within the 95% confidence intervals of the combined results, indicating that there is no statistically significant difference between the fit parameters for short and long microtubules.

Supplementary Table 2. The fitted parameters of the breaking rate for the short and long microtubule populations

Microtubule length	$a = r^*$ (μm)	$b = \ln(\lambda_0 \Delta s \Delta t)$
< 6 μm	2.32 (1.90 to 2.78)	-6.36 (-7.12 to -5.68)
≥ 6 μm	2.42 (2.00 to 2.86)	-6.17 (-6.94 to -5.52)
Combined	2.34 (2.04 to 2.66)	-6.30 (-6.84 to -5.80)

(5) Time dependence of breaking rate

To test if the parameters depend on time of the breaking event, we split the population of microtubules into two equally large groups with one population containing all microtubule segments from microtubules in the first half of each recording ($t < 900$ s) and the other containing all microtubule segment from microtubules in the second half of each recording ($t \geq 900$ s). The same maximum likelihood estimation procedure described in Supplementary Information Section 3 was applied to each population. Part of the MLE procedure includes evaluating the likelihood profile to find the error bounds of the fitted parameters. The parameters and their error are shown in Supplementary Table 3. The calculated parameters for each population are within the 95% confidence intervals of the combined results, which shows that there is no statistically significant difference between the fit parameters for the first and the second half of the experiment, indicating that there is no aging.

Supplementary Table 3. The fitted parameters of the breaking rate for the early and late microtubule populations

Segment appearance	$a = r^*$ (μm)	$b = \ln(\lambda_0 \Delta s \Delta t)$
< 900 s	2.38 (2.00 to 2.78)	-6.18 (-6.84 to -5.60)
≥ 900 s	2.31 (1.77 to 2.87)	-6.49 (-7.43 to -5.65)
Combined	2.34 (2.04 to 2.66)	-6.30 (-6.84 to -5.80)

(6) Location and Timing of Breaking events

Of the 116 observed breaking events, five were from gliding microtubules and 111 were from pinned microtubules. The five gliding microtubules broke 7.0 μm, 1.2 μm, 3.5 μm, 2.3 μm, and 1.2 μm from the leading tip. For the 111 pinned microtubules, the complement of the cumulative distribution of distances between leading tip and breaking site are shown in Supplementary Figure 2a. For the gliding microtubules, all the breaking events occur a significant distance from the tip. In contrast, most of the breaking events for pinned microtubules occur near the tip suggesting that pinning leads to localized stress near the leading tip of the microtubule.

For the 111 pinned microtubules, the complement of the cumulative distribution of the time between the pinning and the breaking event is shown in Supplementary Figure 2b.

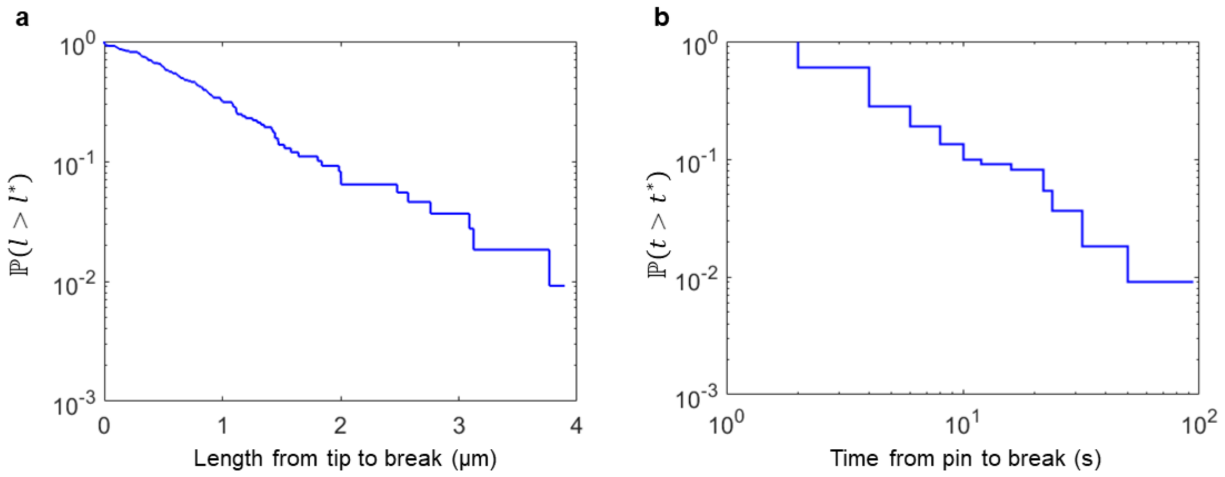

Supplementary Figure 2. (a) Complement of the cumulative distribution function of the distances from the leading tip of the microtubule to the point of breaking across all experiments. (b) Complement of the cumulative distribution function of the time from the start of a pinning event to the breaking of the microtubule.

(7) Alternative explanations for the difference in the breaking rate between smoothly gliding and pinned microtubules: Compression, kinesin interactions, aging

Photodamage: Fluorescently labelled microtubules have been shown to break apart from continuous light exposure on a timescale of minutes at typical excitation intensities in the presence of an antifade system[2,3]. To assess the possibility that photodamage is responsible for breaking events in our system, we compare the exposure time of gliding and pinned microtubules. We randomly selected 40 gliding microtubules (10 from each video) and measured that in average they spent 180 ± 20 s (mean ± SE) in the field-of-view (where they are 10% of the time exposed to light), which is plausible given the size of the field-of-view of 80 μm x 80 μm and a gliding velocity of 0.9 μm/s. In contrast, most pinned microtubules break within 5 s of being pinned, as shown in Supplementary Figure 2b. Therefore, the pinned microtubules do not experience a significantly larger dose of light exposure, and photobleaching cannot account for the 3,000-fold difference in breaking rate.

Lattice defects: While breaking, of course, creates a lattice defect, the role of pre-existing lattice defects in facilitating breaking deserves consideration. Schaedel et al.[4] speculated in 2015 that pre-existing lattice defects (such as sites where the number of protofilaments in a microtubule changes[5,6]) are a source of lattice damage when the microtubule is bent. In 2018, Schaedel et al.[7] investigated the role of lattice defects more broadly, and modeled spontaneous microtubule breaking in a Monte Carlo simulation. They found that (SI, section II): "Microtubules containing defects broke faster than without defects, although the difference was small, and can be attributed rather to the presence of less stable lattice structures than to the presence of the defects themselves, i.e. the 12 protofilament lattice structure breaks faster, than the more stable 13 protofilament lattice structure. Apparently, the perturbation of the lattice structure (i.e. missing bonds at the dislocation)

and the strain accumulated at the defect core was too small to initiate breakage.”. These computational results confirm the intuition about the strain distribution in the microtubule at a transition site from a segment with a larger number of protofilaments to a segment with one less protofilament: The longitudinal forces created by bending are carried across the interface by the continuous protofilaments, while the terminating protofilament does not experience longitudinal forces at the final dimer. Bending is therefore not expected to destabilize the defect site, beyond the introduction of small off-axis stresses on the protofilaments passing the terminating protofilament.

Our experiments support the idea that pre-existing lattice defects are not preferential sites of breaking with two findings: (1) Our measured breaking rate for smoothly gliding microtubules matches the unbinding rate of a tubulin dimer from the intact microtubule lattice calculated with the mechanochemical model of VanBuren et al.[8]This suggest that the removal of a single tubulin dimer initiates breaking and that this dimer does not reside at a preexisting defect. (2) If the uniformly distributed lattice defects would be the breaking sites, we would expect a less pronounced dependence on curvature and the position relative to the end of the tip (Supplementary Figure 2).

Compression: To evaluate the role of compressive forces arising from the motor forces on the gliding microtubules, we have conducted Brownian Dynamics simulations[9,10] of pinned microtubules (Supplementary Figure 4a) as well as microtubules smoothly gliding in circular chambers whose radii are comparable with typical curvatures of pinned microtubules (1.5 and 3.0 μm) for comparison (Supplementary Figure 4b). We found that the compressive forces generated by the kinesins on the lattice increase with the curvature and are identical for smoothly gliding and

pinned microtubules (Supplementary Figure 3c). We conclude that compressive stresses cannot account for the difference between smoothly gliding and pinned microtubules.

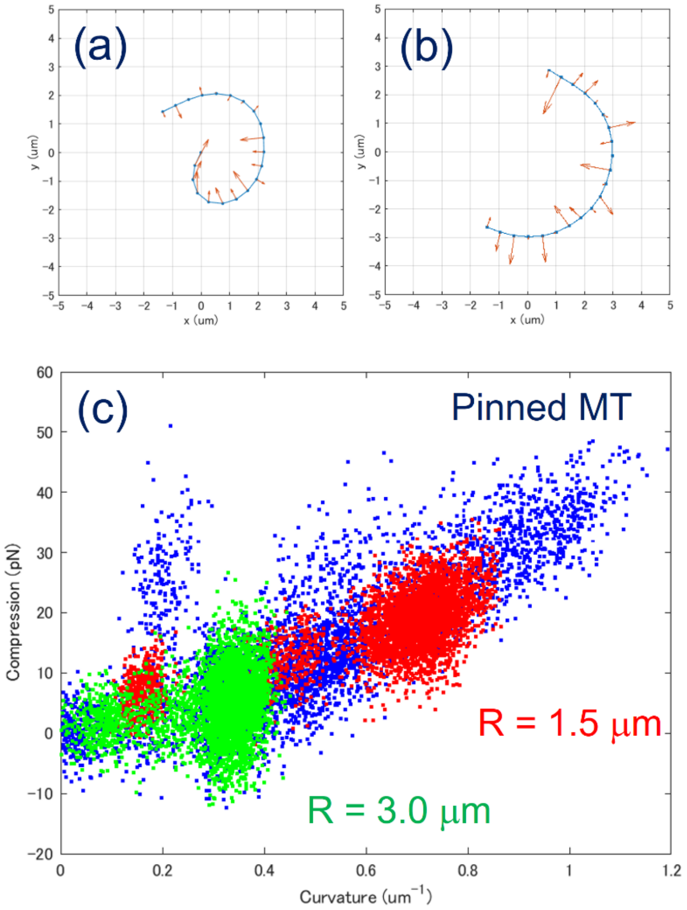

Supplementary Figure 3. (a) Brownian Dynamics simulation of a pinned microtubule (the blue curve). The arrows indicate the magnitude and direction of the kinesin forces on each segment. The leading tip is pinning at the origin. (b) Brownian Dynamics simulation of a microtubule (the blue curve) smoothly gliding in a circular chambers whose radius is 5 μm. The arrows indicate the magnitude and direction of the kinesin forces on each segment. (c) Curvature-compression relation for pinned microtubules (blue dots). For comparison, curvature-compression relations for smoothly gliding microtubules confined within circular chambers with the radii of 1.5 (red dots) and 3.0 (green dots) μm are shown.

Kinesin interactions: The interaction between kinesins and a pinned microtubule are of a peculiar character in the first 1-2 μm behind the pinned tip where most breaking events occur (Supplementary Fig. 2): Within the first micrometer from the attachment point, the kinesins generate load along the microtubule axis and operate at their stall force[11] of 4-7 pN (Supplementary Fig. 3a). For the next five to ten micrometers from the attachment points, the kinesins are pulling perpendicularly to the axis as the buckling microtubule moves sideways. For the rupture force under perpendicular loading, Khataee and Howard[12] give a force-dependent rate equation and associated parameters implying that the force required to unbind kinesin from the microtubule in 30 ms (roughly the time required to stretch the kinesin tail to full length when the microtubule is moving at our velocity of 900 nm/s) is 30 pN. So the forces exerted by the kinesins on their tubulin attachment points is expected to increase (or at least not decrease) with the distance from the attachment point as the force changes from axial loading with the stall force to perpendicular loading with the rupture force. However, the breaking probability rapidly decreases with the distance from the attachment point, which is inconsistent with the force profile along the microtubule.

Triclin et al.[13] propose that already the internal forces exerted by kinesin walking along microtubules can enhance the removal of tubulins from the lattice five-fold. Kuo et al.[14] similarly measured that a single kinesin pulling with its stall force (4-7 pN) accelerates tubulin removal about five-fold. Thus, kinesin forces enhance the probability of tubulin removal, but are not the main factor responsible for the distribution of breaking sites and the acceleration of breaking.

Aging: Another potential explanation for the difference between smoothly gliding and pinned microtubules is that the segments of a smoothly gliding microtubule quickly pass through a trajectory region with high curvature, while the segments of a pinned microtubule remain highly

curved for an extended time. If breaking is facilitated by a recent history of high curvature (causing "aging"), this could increase the breaking probability for pinned microtubules. We tested this by reviewing the recent history of breaking events. For each breaking event, we compared the curvature during the breaking event to the curvature of the same microtubule segment in the preceding frame (Supplementary Figure 4a) and the average curvature of the 5 previous frames (Supplementary Figure 4b). Many of the segments experiencing a high curvature breaking event (>1 μm^{-1}) have lower curvatures in the preceding frames, comparable to the average curvature of gliding microtubules of 0.12 ± 0.14 μm^{-1}. This implies that breaking is not dependent on a recent history of bending. To further investigate this, the population of breaking events was split into two subpopulations: breaking events where the curvature in the previous frame was larger than 0.3 times the breaking curvature and events where the curvature in the previous frame was smaller than 0.3 times the breaking curvature. The breaking curvatures of the two populations were compared using a two-sample t-test and no significant difference was found ($p = 0.18$). If the analysis is repeated with two populations, where one population comprises the breaking events where the average curvature in the previous five frames was greater than 0.3 times the breaking curvature AND the breaking curvature was at least 0.5 μm^{-1}, and the other population comprises the breaking events where the average curvature in the previous five frames was less than 0.3 times the breaking curvature AND the breaking curvature was at least 0.5 μm^{-1}, we found again no statistical difference ($p = 0.52$). Thus, a recent history of bending does not affect the probability of breaking, i.e. there is no aging of the microtubules.

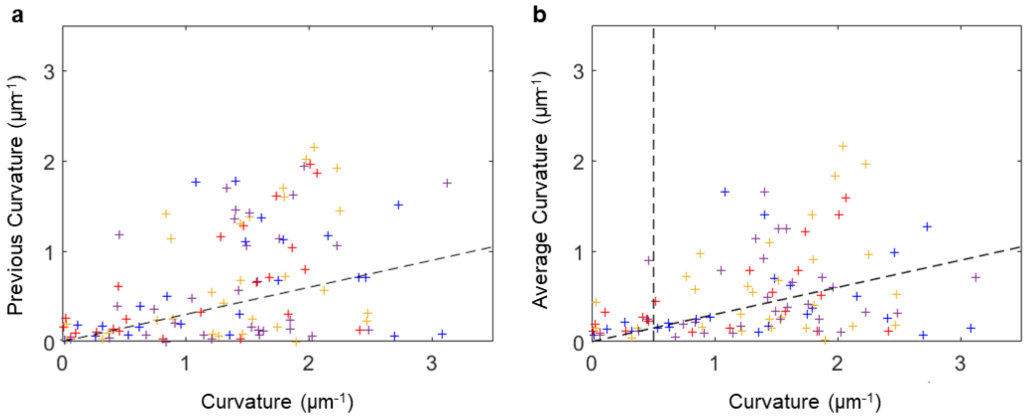

Supplementary Figure 4. Curvature of breaking segments at the moment of breaking versus (a) the curvature of the previous frame and (b) the average curvature of the previous five frames. (Experiment 1: N=25 – blue; Exp. 2: N=22 – yellow; Exp. 3: N=32 – red, Exp. 4: N=32 – purple).

(8) Equilibrium Curvature Distribution Due to Breaking

The aim of this calculation is to determine the steady state curvature distribution of a population of microtubules which is subject to a curvature-reducing process and a curvature-producing process. The curvature-reducing process is breaking, which removes segments with a given curvature with a rate that exponentially increases with the segments curvature. The curvature-producing process is either the extension of the segment length resulting from motor propulsion present in our experiments (Supplementary Figure 5a) or the compression of the segment end points resulting from intracellular flows as described by Bicek et al.[15] (Supplementary Figure 5b). In this way we aim to model the curvature distribution for curvatures above 0.3 μm^{-1}, where the thermal fluctuations make only a small contributions due to the high stiffness of the microtubules.

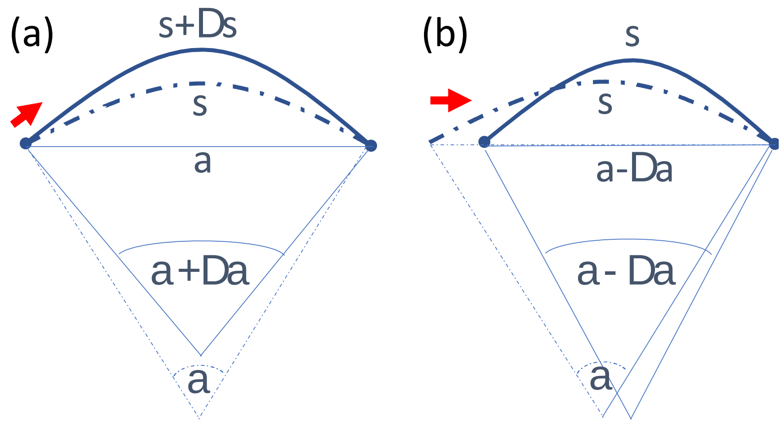

Supplementary Figure 5. Curvature-producing processes. (a) A segment with arc length s may experience an increase in length while its end points are fixed at a distance a (e.g. because the end points are motors moving a microtubule at different velocities). (b) A segment with fixed arc length s may experience a reduction of the distance a between its end points (e.g. due to retrograde flow compressing a microtubule).

If we consider a microtubule segment as a circular arc segment between two attachment points, the distance between the two points, a, can be described by the radius R and the arc angle α:

$$a = 2Rsin(\alpha/2) = R\alpha - R\frac{\alpha^3}{24} \tag{1}$$

The length of the microtubule segment is equivalent to the arc length of the osculating circle:

$$s = R\alpha \tag{2}$$

Increasing the arc length by Δs while maintaining the distance between the attachment points leads to a relationship between the radius of curvature before (R) and after (R- ΔR) the increase in the arc length:

$$a \; = \; (s + \Delta s) - \frac{(s + \Delta s)^3}{24(R - \Delta R)^2} = s - \frac{s^3}{24R^2} \tag{3}$$

If the radius of curvature is expressed as the inverse of the curvature κ, eq. (3) can be rewritten as:

$$s + \Delta s - (\kappa + \Delta\kappa)^2 \frac{(s + \Delta s)^3}{24} = s - \kappa^2 \frac{s^3}{24} \tag{4}$$

Rearranging and neglecting higher order terms yields:

$$-\Delta s + \kappa^2 \frac{(s + \Delta s)^3}{24} + 2\kappa\Delta\kappa \frac{(s + \Delta s)^3}{24} = \kappa^2 \frac{s^3}{24} \tag{5}$$

$$\Delta\kappa = \Delta s \frac{12}{\kappa s^3} \tag{6}$$

Alternatively, decreasing the distance a between the attachment points by Δa while maintaining the arc length s yields from (1) and (2):

$$\Delta a = -\kappa^2 \frac{s^3}{24} + (\kappa + \Delta\kappa)^2 \frac{s^3}{24} \tag{7}$$

After neglecting higher order terms and using Δa ≈ Δs, equation (6) is again obtained:

$$\Delta s = \Delta\kappa \frac{s^3\kappa}{12} \tag{8}$$

If we define the compression velocity v as $v = ds/dt$, an expression for the rate of curvature production is obtained:

$$\frac{d\kappa}{dt} = \frac{12v}{\kappa s^3} \tag{9}$$

This process shifts the curvature distribution f(κ,t) towards higher curvatures and the breaking rate λ(κ) acts on the curvature distribution by removing segments of high curvature according to:

$$f(\kappa, t + dt) = (1 - \lambda(\kappa)dt)f(\kappa - d\kappa, t) = f(\kappa, t) - \frac{df(\kappa, t)}{d\kappa}d\kappa - \lambda(\kappa)dt \times f(\kappa, t) \tag{10}$$

If a steady state is reached, $f(\kappa, t + dt) = f(\kappa, t)$, so that:

$$\frac{df(\kappa, t)}{d\kappa}d\kappa = -\lambda(\kappa)dt \times f(\kappa, t) \tag{11}$$

Which yields with Equations (6,8) and the equation for the curvature-dependent breaking rate:

$$\frac{df(\kappa)}{f(\kappa)} = \frac{-\kappa s^3\lambda_0 exp(\kappa r^*)}{12v}d\kappa \tag{12}$$

Integration yields the steady state distribution:

$$f(\kappa) = f(0)exp\left(-\frac{s^3\lambda_0}{12v(r^*)^2}(\kappa r^* exp(\kappa r^*) - exp(\kappa r^*) - 1)\right) \tag{13}$$

Supplementary Figure 6 shows the fit of Equation (13) with a compression velocity of v = 0.51 nm/s to the curvature distribution of the pinned microtubules above 0.3 μm^{-1}. Equation (13) does not account for thermal fluctuations which determine the curvature distribution up until 0.3 μm^{-1} as shown in Figure 3a of the main text.

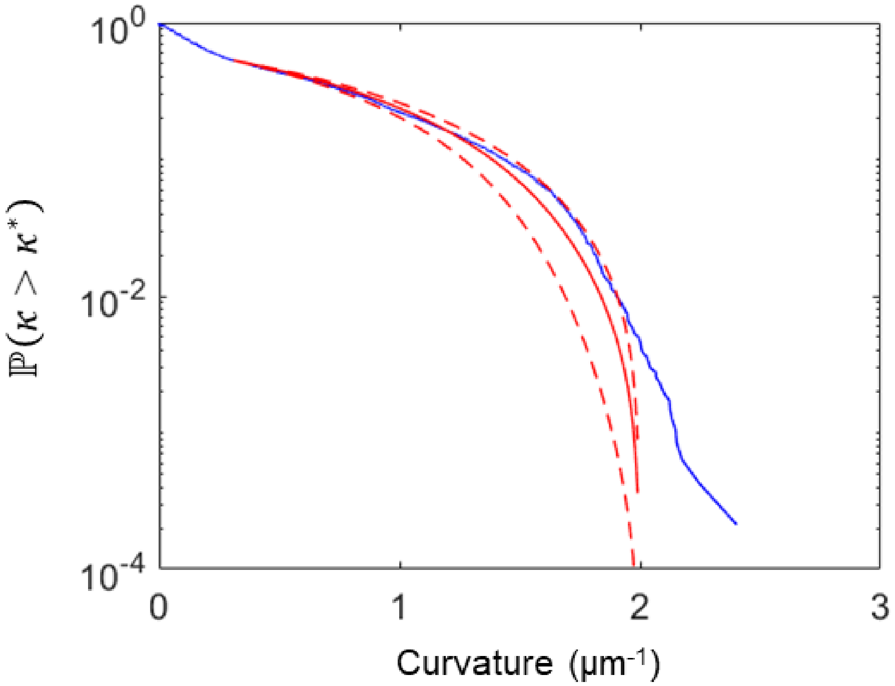

Supplementary Figure 6. The steady state curvature distribution (solid red line) fitted to the observed pinned microtubule curvature distribution (blue line). The confidence interval of the steady state curvature distribution (dotted red line) is determined by evaluating Equation 13 using the 95% confidence interval of the characteristic breaking radius, r*.

Supplementary Videos (Captions)

Supplementary Video 1-4 | Gliding Motility Assays.

Videos of the gliding assays of fluorescently labeled microtubules gliding on surfaces coated with kinesin-1. Each video number corresponds to the experiment number referenced in the main text (Figures 3 and 4). Video speed: 50x accelerated relative to real time. Field of View: 133.12 μm x 133.12 μm.

Supplementary Data (Captions)

Supplementary Data 1 | Pinned Microtubule Location.

Excel file containing data on each manually selected nonbreaking pinned microtubule. Included is the location of the pinned tip and the frame the microtubules become pinned and unpinned. Data are separated into sheets corresponding to the experiment number referenced in the main text (Figures 3 and 4).

Supplementary Data 2 | Breaking Microtubule Location

Excel file containing data on each manually selected breaking microtubule. Included is the breaking point of the microtubule, the frame in which the break occurs, whether the break was the result of a pinning event and how long the microtubule was pinned before the breaking event. Data are separated into sheets corresponding to the experiment number referenced in the main text (Figures 3 and 4).

Supplementary References

S1. Nitta, T., Tanahashi, A., Hirano, M. & Hess, H. Simulating molecular shuttle movements: Towards computer-aided design of nanoscale transport systems. *Lab Chip* **6**, 881–885 (2006).

S2. Vigers, G. P., Coue, M. & McIntosh, J. R. Fluorescent microtubules break up under illumination. *J. Cell Biol.* **107**, 1011–1024 (1988).

S3. Brunner, C., Ernst, K.-H., Hess, H. & Vogel, V. Lifetime of biomolecules in polymer-based hybrid nanodevices. *Nanotechnology* **15**, S540–S548 (2004).

S4. Schaedel, L. *et al.* Microtubules self-repair in response to mechanical stress. *Nat. Mater.* **14**, 1156–1163 (2015).

S5. Reid, T. A., Coombes, C. & Gardner, M. K. Manipulation and quantification of microtubule lattice integrity. *Biol. Open* **6**, 1245–1256 (2017).

S6. Rai, A. *et al.* Lattice defects induced by microtubule-stabilizing agents exert a long-range effect on microtubule growth by promoting catastrophes. *Proc. Natl. Acad. Sci. U. S. A.* **118**, e2112261118 (2021).

S7. Schaedel, L. *et al.* Lattice defects induce microtubule self-renewal. *Nat. Phys.* **15**, 830–838 (2019).

S8. VanBuren, V., Cassimeris, L. & Odde, D. J. Mechanochemical Model of Microtubule Structure and Self-Assembly Kinetics. *Biophys. J.* **89**, 2911–2926 (2005).

S9. Sweet, M., Kang'iri, S. M. & Nitta, T. Linking path and filament persistence lengths of microtubules gliding over kinesin. *Sci. Rep.* **12**, 3081 (2022).

S10. Ishigure, Y. & Nitta, T. Understanding the Guiding of Kinesin/Microtubule-Based Microtransporters in Microfabricated Tracks. *Langmuir* **30**, 12089–12096 (2014).

S11. Howard, J. Mechanics of motor proteins and the cytoskeleton. *(Sinauer Associates Incorporated, 2001)*.

S12. Khataee, H. & Howard, J. Force Generated by Two Kinesin Motors Depends on the Load Direction and Intermolecular Coupling. *Phys. Rev. Lett.* **122**, 188101 (2019).

S13. Triclin, S. *et al.* Self-repair protects microtubules from destruction by molecular motors. *Nat. Mater.* **20**, 883–891 (2021).

S14. Kuo, Y. W., Mahamdeh, M., Tuna, Y. & Howard, J. The force required to remove tubulin from the microtubule lattice by pulling on its α-tubulin C-terminal tail. *Nat. Commun.* **13**, 3651 (2022).

S15. Bicek, A. D. *et al.* Anterograde microtubule transport drives microtubule bending in LLC-PK1 epithelial cells. *Mol. Biol. Cell* **20**, 2943–2953 (2009).
